# Supplementary material for: Artificial Neural Network analysis on the effect of mixed convection in triangular-shaped geometry using water-based Al2O3 nanofluid
Source: PLoS One. 2024 Sep 13;19(9):e0304826. doi: 10.1371/journal.pone.0304826 (PMC11398699; doi:10.1371/journal.pone.0304826)
Supplement: S1 Table — (DOCX) [file pone.0304826.s001.docx]

Supplementary information file:

Forecasted average Nusselt number (Nu_pred_) and given average Nusselt number (Nu_avg_)

| **Ri** | **Ha** | **phi** | **Re** | Nu_avg_ | Nu_pred_ | **Error** |
| --- | --- | --- | --- | --- | --- | --- |
| 0.01 | 0 | 0.01 | 100 | 1.13358920 | 1.134760728 | -0.00117 |
| 0.01 | 0 | 0.01 | 200 | 1.33156871 | 1.331351886 | 0.000217 |
| 0.01 | 0 | 0.01 | 300 | 1.56502187 | 1.564918465 | 0.000103 |
| 0.01 | 0 | 0.01 | 400 | 1.77549923 | 1.775557819 | -5.86e-05 |
| 0.01 | 0 | 0.02 | 100 | 1.15935672 | 1.160081771 | -0.00073 |
| 0.01 | 0 | 0.02 | 200 | 1.35185080 | 1.352774033 | -0.00092 |
| 0.01 | 0 | 0.02 | 300 | 1.57762133 | 1.577194863 | 0.000426 |
| 0.01 | 0 | 0.02 | 400 | 1.79011333 | 1.789906763 | 0.000207 |
| 0.01 | 0 | 0.03 | 100 | 1.18570078 | 1.185378258 | 0.000323 |
| 0.01 | 0 | 0.03 | 200 | 1.37283248 | 1.373868955 | -0.00104 |
| 0.01 | 0 | 0.03 | 300 | 1.58709229 | 1.587316395 | -0.00022 |
| 0.01 | 0 | 0.03 | 400 | 1.80096408 | 1.801114914 | -0.00015 |
| 0.01 | 0 | 0.04 | 100 | 1.21269731 | 1.210521096 | 0.002176 |
| 0.01 | 0 | 0.04 | 200 | 1.39536238 | 1.394277023 | 0.001085 |
| 0.01 | 0 | 0.04 | 300 | 1.59529612 | 1.595388752 | -9.26e-05 |
| 0.01 | 0 | 0.04 | 400 | 1.80902791 | 1.809016225 | 1.17e-05 |
| 0.01 | 20 | 0.01 | 100 | 1.22359021 | 1.224147673 | -0.00056 |
| 0.01 | 20 | 0.01 | 200 | 1.44553760 | 1.444307835 | 0.00123 |
| 0.01 | 20 | 0.01 | 300 | 1.61463306 | 1.614488120 | 0.000145 |
| 0.01 | 20 | 0.01 | 400 | 1.77403055 | 1.775078980 | -0.00105 |
| 0.01 | 20 | 0.02 | 100 | 1.24916387 | 1.249604377 | -0.00044 |
| 0.01 | 20 | 0.02 | 200 | 1.47376340 | 1.473303858 | 0.00046 |
| 0.01 | 20 | 0.02 | 300 | 1.64534267 | 1.644750782 | 0.000592 |
| 0.01 | 20 | 0.02 | 400 | 1.80647745 | 1.806153599 | 0.000324 |
| 0.01 | 20 | 0.03 | 100 | 1.27471049 | 1.274600091 | 0.00011 |
| 0.01 | 20 | 0.03 | 200 | 1.50151876 | 1.502243262 | -0.00072 |
| 0.01 | 20 | 0.03 | 300 | 1.67481448 | 1.674526738 | 0.000288 |
| 0.01 | 20 | 0.03 | 400 | 1.83644963 | 1.835816160 | 0.000633 |
| 0.01 | 20 | 0.04 | 100 | 1.30015836 | 1.298984779 | 0.001174 |
| 0.01 | 20 | 0.04 | 200 | 1.52870251 | 1.530876622 | -0.00217 |
| 0.01 | 20 | 0.04 | 300 | 1.70297470 | 1.703835356 | -0.00086 |
| 0.01 | 20 | 0.04 | 400 | 1.86405042 | 1.863738376 | 0.000312 |
| 0.01 | 50 | 0.01 | 100 | 1.18927565 | 1.187323278 | 0.001952 |
| 0.01 | 50 | 0.01 | 200 | 1.42542185 | 1.426269060 | -0.00085 |
| 0.01 | 50 | 0.01 | 300 | 1.58943238 | 1.590096628 | -0.00066 |
| 0.01 | 50 | 0.01 | 400 | 1.71749220 | 1.716641559 | 0.000851 |
| 0.01 | 50 | 0.02 | 100 | 1.21903280 | 1.218519721 | 0.000513 |
| 0.01 | 50 | 0.02 | 200 | 1.45858792 | 1.459038596 | -0.00045 |
| 0.01 | 50 | 0.02 | 300 | 1.62539998 | 1.625902401 | -0.0005 |
| 0.01 | 50 | 0.02 | 400 | 1.75575505 | 1.755841370 | -8.63e-05 |
| 0.01 | 50 | 0.03 | 100 | 1.24959260 | 1.250257054 | -0.00066 |
| 0.01 | 50 | 0.03 | 200 | 1.49261943 | 1.492261624 | 0.000358 |
| 0.01 | 50 | 0.03 | 300 | 1.66232118 | 1.661916000 | 0.000405 |
| 0.01 | 50 | 0.03 | 400 | 1.79506975 | 1.795403307 | -0.00033 |
| 0.01 | 50 | 0.04 | 100 | 1.28071027 | 1.282526349 | -0.00182 |
| 0.01 | 50 | 0.04 | 200 | 1.52727785 | 1.525788293 | 0.00149 |
| 0.01 | 50 | 0.04 | 300 | 1.69993164 | 1.698462726 | 0.001469 |
| 0.01 | 50 | 0.04 | 400 | 1.83515650 | 1.835098882 | 5.76e-05 |
| 0.01 | 100 | 0.01 | 100 | 1.08327216 | 1.083068887 | 0.000203 |
| 0.01 | 100 | 0.01 | 200 | 1.34625801 | 1.345976310 | 0.000282 |
| 0.01 | 100 | 0.01 | 300 | 1.52509942 | 1.523773634 | 0.001326 |
| 0.01 | 100 | 0.01 | 400 | 1.66308252 | 1.663710562 | -0.00063 |
| 0.01 | 100 | 0.02 | 100 | 1.11624361 | 1.116647171 | -0.0004 |
| 0.01 | 100 | 0.02 | 200 | 1.38233784 | 1.382641609 | -0.0003 |
| 0.01 | 100 | 0.02 | 300 | 1.56311140 | 1.562916467 | 0.000195 |
| 0.01 | 100 | 0.02 | 400 | 1.70302780 | 1.703388503 | -0.00036 |
| 0.01 | 100 | 0.03 | 100 | 1.15073734 | 1.150844067 | -0.00011 |
| 0.01 | 100 | 0.03 | 200 | 1.41966917 | 1.419875999 | -0.00021 |
| 0.01 | 100 | 0.03 | 300 | 1.60232103 | 1.602513623 | -0.00019 |
| 0.01 | 100 | 0.03 | 400 | 1.74420376 | 1.743985596 | 0.000218 |
| 0.01 | 100 | 0.04 | 100 | 1.18627288 | 1.185662544 | 0.00061 |
| 0.01 | 100 | 0.04 | 200 | 1.45779090 | 1.457637873 | 0.000153 |
| 0.01 | 100 | 0.04 | 300 | 1.64234239 | 1.642642414 | -0.0003 |
| 0.01 | 100 | 0.04 | 400 | 1.78625597 | 1.785390842 | 0.000865 |
| 0.1 | 0 | 0.01 | 100 | 1.13358920 | 1.134762764 | -0.00117 |
| 0.1 | 0 | 0.01 | 200 | 1.33156871 | 1.331350975 | 0.000218 |
| 0.1 | 0 | 0.01 | 300 | 1.56502189 | 1.564904428 | 0.000117 |
| 0.1 | 0 | 0.01 | 400 | 1.77549925 | 1.775551310 | -5.21e-05 |
| 0.1 | 0 | 0.02 | 100 | 1.15935672 | 1.160082779 | -0.00073 |
| 0.1 | 0 | 0.02 | 200 | 1.35185080 | 1.352765572 | -0.00091 |
| 0.1 | 0 | 0.02 | 300 | 1.57762135 | 1.577173403 | 0.000448 |
| 0.1 | 0 | 0.02 | 400 | 1.79011335 | 1.789905296 | 0.000208 |
| 0.1 | 0 | 0.03 | 100 | 1.18570078 | 1.185377365 | 0.000323 |
| 0.1 | 0 | 0.03 | 200 | 1.37283248 | 1.373836253 | -0.001 |
| 0.1 | 0 | 0.03 | 300 | 1.58709231 | 1.587315641 | -0.00022 |
| 0.1 | 0 | 0.03 | 400 | 1.80096410 | 1.801111911 | -0.00015 |
| 0.1 | 0 | 0.04 | 100 | 1.21269731 | 1.210512461 | 0.002185 |
| 0.1 | 0 | 0.04 | 200 | 1.39536238 | 1.394243722 | 0.001119 |
| 0.1 | 0 | 0.04 | 300 | 1.59529614 | 1.595390819 | -9.47e-05 |
| 0.1 | 0 | 0.04 | 400 | 1.80902793 | 1.809010888 | 1.70e-05 |
| 0.1 | 20 | 0.01 | 100 | 1.22359021 | 1.224158866 | -0.00057 |
| 0.1 | 20 | 0.01 | 200 | 1.44553760 | 1.444308127 | 0.001229 |
| 0.1 | 20 | 0.01 | 300 | 1.61463306 | 1.614477120 | 0.000156 |
| 0.1 | 20 | 0.01 | 400 | 1.77403055 | 1.775075085 | -0.00104 |
| 0.1 | 20 | 0.02 | 100 | 1.24916387 | 1.249616344 | -0.00045 |
| 0.1 | 20 | 0.02 | 200 | 1.47376340 | 1.473300668 | 0.000463 |
| 0.1 | 20 | 0.02 | 300 | 1.64534267 | 1.644719009 | 0.000624 |
| 0.1 | 20 | 0.02 | 400 | 1.80647745 | 1.806160836 | 0.000317 |
| 0.1 | 20 | 0.03 | 100 | 1.27471049 | 1.274612589 | 9.79e-05 |
| 0.1 | 20 | 0.03 | 200 | 1.50151876 | 1.502223454 | -0.0007 |
| 0.1 | 20 | 0.03 | 300 | 1.67481448 | 1.674516129 | 0.000298 |
| 0.1 | 20 | 0.03 | 400 | 1.83644963 | 1.835823449 | 0.000626 |
| 0.1 | 20 | 0.04 | 100 | 1.30015836 | 1.298994708 | 0.001164 |
| 0.1 | 20 | 0.04 | 200 | 1.52870251 | 1.530842000 | -0.00214 |
| 0.1 | 20 | 0.04 | 300 | 1.70297470 | 1.703833307 | -0.00086 |
| 0.1 | 20 | 0.04 | 400 | 1.86405043 | 1.863743671 | 0.000307 |
| 0.1 | 50 | 0.01 | 100 | 1.18927565 | 1.187333517 | 0.001942 |
| 0.1 | 50 | 0.01 | 200 | 1.42542185 | 1.426275543 | -0.00085 |
| 0.1 | 50 | 0.01 | 300 | 1.58943239 | 1.590102020 | -0.00067 |
| 0.1 | 50 | 0.01 | 400 | 1.71749220 | 1.716626113 | 0.000866 |
| 0.1 | 50 | 0.02 | 100 | 1.21903280 | 1.218529529 | 0.000503 |
| 0.1 | 50 | 0.02 | 200 | 1.45858792 | 1.459043385 | -0.00046 |
| 0.1 | 50 | 0.02 | 300 | 1.62539998 | 1.625884219 | -0.00048 |
| 0.1 | 50 | 0.02 | 400 | 1.75575505 | 1.755847626 | -9.26e-05 |
| 0.1 | 50 | 0.03 | 100 | 1.24959260 | 1.250266263 | -0.00067 |
| 0.1 | 50 | 0.03 | 200 | 1.49261943 | 1.492258170 | 0.000361 |
| 0.1 | 50 | 0.03 | 300 | 1.66232118 | 1.661902669 | 0.000419 |
| 0.1 | 50 | 0.03 | 400 | 1.79506975 | 1.795414384 | -0.00034 |
| 0.1 | 50 | 0.04 | 100 | 1.28071027 | 1.282533684 | -0.00182 |
| 0.1 | 50 | 0.04 | 200 | 1.52727785 | 1.525760389 | 0.001517 |
| 0.1 | 50 | 0.04 | 300 | 1.69993164 | 1.698467926 | 0.001464 |
| 0.1 | 50 | 0.04 | 400 | 1.83515650 | 1.835110201 | 4.63e-05 |
| 0.1 | 100 | 0.01 | 100 | 1.08327216 | 1.083081571 | 0.000191 |
| 0.1 | 100 | 0.01 | 200 | 1.34625801 | 1.345995035 | 0.000263 |
| 0.1 | 100 | 0.01 | 300 | 1.52509942 | 1.523792442 | 0.001307 |
| 0.1 | 100 | 0.01 | 400 | 1.66308253 | 1.663683646 | -0.0006 |
| 0.1 | 100 | 0.02 | 100 | 1.11624361 | 1.116659557 | -0.00042 |
| 0.1 | 100 | 0.02 | 200 | 1.38233784 | 1.382659433 | -0.00032 |
| 0.1 | 100 | 0.02 | 300 | 1.56311140 | 1.562926298 | 0.000185 |
| 0.1 | 100 | 0.02 | 400 | 1.70302780 | 1.703370999 | -0.00034 |
| 0.1 | 100 | 0.03 | 100 | 1.15073734 | 1.150856123 | -0.00012 |
| 0.1 | 100 | 0.03 | 200 | 1.41966917 | 1.419891459 | -0.00022 |
| 0.1 | 100 | 0.03 | 300 | 1.60232103 | 1.602499411 | -0.00018 |
| 0.1 | 100 | 0.03 | 400 | 1.74420376 | 1.743986105 | 0.000218 |
| 0.1 | 100 | 0.04 | 100 | 1.18627288 | 1.185674016 | 0.000599 |
| 0.1 | 100 | 0.04 | 200 | 1.45779090 | 1.457641619 | 0.000149 |
| 0.1 | 100 | 0.04 | 300 | 1.64234239 | 1.642643712 | -0.0003 |
| 0.1 | 100 | 0.04 | 400 | 1.78625597 | 1.785394797 | 0.000861 |
| 1 | 0 | 0.01 | 100 | 1.13358921 | 1.134782676 | -0.00119 |
| 1 | 0 | 0.01 | 200 | 1.33156878 | 1.331324004 | 0.000245 |
| 1 | 0 | 0.01 | 300 | 1.56502207 | 1.564679470 | 0.000343 |
| 1 | 0 | 0.01 | 400 | 1.77549948 | 1.775537519 | -3.80e-05 |
| 1 | 0 | 0.02 | 100 | 1.15935672 | 1.160089649 | -0.00073 |
| 1 | 0 | 0.02 | 200 | 1.35185086 | 1.352585416 | -0.00073 |
| 1 | 0 | 0.02 | 300 | 1.57762153 | 1.577089646 | 0.000532 |
| 1 | 0 | 0.02 | 400 | 1.79011358 | 1.789897830 | 0.000216 |
| 1 | 0 | 0.03 | 100 | 1.18570078 | 1.185344575 | 0.000356 |
| 1 | 0 | 0.03 | 200 | 1.37283252 | 1.373463192 | -0.00063 |
| 1 | 0 | 0.03 | 300 | 1.58709248 | 1.587347657 | -0.00026 |
| 1 | 0 | 0.03 | 400 | 1.80096432 | 1.801082539 | -0.00012 |
| 1 | 0 | 0.04 | 100 | 1.21269731 | 1.210315126 | 0.002382 |
| 1 | 0 | 0.04 | 200 | 1.39536241 | 1.394035620 | 0.001327 |
| 1 | 0 | 0.04 | 300 | 1.59529629 | 1.595416833 | -0.00012 |
| 1 | 0 | 0.04 | 400 | 1.80902815 | 1.808957342 | 7.08e-05 |
| 1 | 20 | 0.01 | 100 | 1.22359022 | 1.224270405 | -0.00068 |
| 1 | 20 | 0.01 | 200 | 1.44553761 | 1.444301371 | 0.001236 |
| 1 | 20 | 0.01 | 300 | 1.61463308 | 1.614240440 | 0.000393 |
| 1 | 20 | 0.01 | 400 | 1.77403058 | 1.775119883 | -0.00109 |
| 1 | 20 | 0.02 | 100 | 1.24916387 | 1.249734160 | -0.00057 |
| 1 | 20 | 0.02 | 200 | 1.47376340 | 1.473206580 | 0.000557 |
| 1 | 20 | 0.02 | 300 | 1.64534268 | 1.644506963 | 0.000836 |
| 1 | 20 | 0.02 | 400 | 1.80647748 | 1.806247201 | 0.00023 |
| 1 | 20 | 0.03 | 100 | 1.27471049 | 1.274724461 | -1.40e-05 |
| 1 | 20 | 0.03 | 200 | 1.50151876 | 1.501911388 | -0.00039 |
| 1 | 20 | 0.03 | 300 | 1.67481450 | 1.674477413 | 0.000337 |
| 1 | 20 | 0.03 | 400 | 1.83644965 | 1.835898049 | 0.000552 |
| 1 | 20 | 0.04 | 100 | 1.30015837 | 1.299016520 | 0.001142 |
| 1 | 20 | 0.04 | 200 | 1.52870252 | 1.530620891 | -0.00192 |
| 1 | 20 | 0.04 | 300 | 1.70297471 | 1.703823063 | -0.00085 |
| 1 | 20 | 0.04 | 400 | 1.86405045 | 1.863796742 | 0.000254 |
| 1 | 50 | 0.01 | 100 | 1.18927565 | 1.187435782 | 0.00184 |
| 1 | 50 | 0.01 | 200 | 1.42542185 | 1.426336595 | -0.00091 |
| 1 | 50 | 0.01 | 300 | 1.58943239 | 1.590052404 | -0.00062 |
| 1 | 50 | 0.01 | 400 | 1.71749222 | 1.716600958 | 0.000891 |
| 1 | 50 | 0.02 | 100 | 1.21903280 | 1.218626948 | 0.000406 |
| 1 | 50 | 0.02 | 200 | 1.45858792 | 1.459063854 | -0.00048 |
| 1 | 50 | 0.02 | 300 | 1.62539999 | 1.625677997 | -0.00028 |
| 1 | 50 | 0.02 | 400 | 1.75575506 | 1.755945143 | -0.00019 |
| 1 | 50 | 0.03 | 100 | 1.24959260 | 1.250353355 | -0.00076 |
| 1 | 50 | 0.03 | 200 | 1.49261944 | 1.492106080 | 0.000513 |
| 1 | 50 | 0.03 | 300 | 1.66232119 | 1.661888317 | 0.000433 |
| 1 | 50 | 0.03 | 400 | 1.79506976 | 1.795529996 | -0.00046 |
| 1 | 50 | 0.04 | 100 | 1.28071027 | 1.282571140 | -0.00186 |
| 1 | 50 | 0.04 | 200 | 1.52727785 | 1.525503311 | 0.001775 |
| 1 | 50 | 0.04 | 300 | 1.69993164 | 1.698546195 | 0.001385 |
| 1 | 50 | 0.04 | 400 | 1.83515651 | 1.835224064 | -6.76e-05 |
| 1 | 100 | 0.01 | 100 | 1.08327216 | 1.083208378 | 6.38e-05 |
| 1 | 100 | 0.01 | 200 | 1.34625801 | 1.346181398 | 7.66e-05 |
| 1 | 100 | 0.01 | 300 | 1.52509942 | 1.523949234 | 0.00115 |
| 1 | 100 | 0.01 | 400 | 1.66308253 | 1.663413131 | -0.00033 |
| 1 | 100 | 0.02 | 100 | 1.11624361 | 1.116783275 | -0.00054 |
| 1 | 100 | 0.02 | 200 | 1.38233784 | 1.382831758 | -0.00049 |
| 1 | 100 | 0.02 | 300 | 1.56311140 | 1.562901804 | 0.00021 |
| 1 | 100 | 0.02 | 400 | 1.70302780 | 1.703308182 | -0.00028 |
| 1 | 100 | 0.03 | 100 | 1.15073734 | 1.150975681 | -0.00024 |
| 1 | 100 | 0.03 | 200 | 1.41966917 | 1.420005064 | -0.00034 |
| 1 | 100 | 0.03 | 300 | 1.60232103 | 1.602401625 | -8.06e-05 |
| 1 | 100 | 0.03 | 400 | 1.74420376 | 1.744014316 | 0.000189 |
| 1 | 100 | 0.04 | 100 | 1.18627288 | 1.185780953 | 0.000492 |
| 1 | 100 | 0.04 | 200 | 1.45779090 | 1.457550619 | 0.00024 |
| 1 | 100 | 0.04 | 300 | 1.64234240 | 1.642752380 | -0.00041 |
| 1 | 100 | 0.04 | 400 | 1.78625598 | 1.785437482 | 0.000818 |
| 5 | 0 | 0.01 | 100 | 1.13358922 | 1.134270704 | -0.00068 |
| 5 | 0 | 0.01 | 200 | 1.33156907 | 1.330639897 | 0.000929 |
| 5 | 0 | 0.01 | 300 | 1.56502290 | 1.564873072 | 0.00015 |
| 5 | 0 | 0.01 | 400 | 1.77550049 | 1.775633776 | -0.00013 |
| 5 | 0 | 0.02 | 100 | 1.15935673 | 1.159447712 | -9.10e-05 |
| 5 | 0 | 0.02 | 200 | 1.35185109 | 1.351947181 | -9.61e-05 |
| 5 | 0 | 0.02 | 300 | 1.57762234 | 1.577410842 | 0.000211 |
| 5 | 0 | 0.02 | 400 | 1.79011457 | 1.789880549 | 0.000234 |
| 5 | 0 | 0.03 | 100 | 1.18570078 | 1.184680510 | 0.00102 |
| 5 | 0 | 0.03 | 200 | 1.37283270 | 1.373059601 | -0.00023 |
| 5 | 0 | 0.03 | 300 | 1.58709326 | 1.587606454 | -0.00051 |
| 5 | 0 | 0.03 | 400 | 1.80096531 | 1.800948734 | 1.66e-05 |
| 5 | 0 | 0.04 | 100 | 1.21269731 | 1.209795842 | 0.002901 |
| 5 | 0 | 0.04 | 200 | 1.39536254 | 1.393676628 | 0.001686 |
| 5 | 0 | 0.04 | 300 | 1.59529700 | 1.595544298 | -0.00025 |
| 5 | 0 | 0.04 | 400 | 1.80902913 | 1.808713266 | 0.000316 |
| 5 | 20 | 0.01 | 100 | 1.22359022 | 1.224236414 | -0.00065 |
| 5 | 20 | 0.01 | 200 | 1.44553764 | 1.443639093 | 0.001899 |
| 5 | 20 | 0.01 | 300 | 1.61463314 | 1.614091224 | 0.000542 |
| 5 | 20 | 0.01 | 400 | 1.77403069 | 1.775610628 | -0.00158 |
| 5 | 20 | 0.02 | 100 | 1.24916388 | 1.249583812 | -0.00042 |
| 5 | 20 | 0.02 | 200 | 1.47376343 | 1.472604246 | 0.001159 |
| 5 | 20 | 0.02 | 300 | 1.64534274 | 1.644561103 | 0.000782 |
| 5 | 20 | 0.02 | 400 | 1.80647758 | 1.806670331 | -0.00019 |
| 5 | 20 | 0.03 | 100 | 1.27471049 | 1.274611848 | 9.86e-05 |
| 5 | 20 | 0.03 | 200 | 1.50151879 | 1.501593189 | -7.44e-05 |
| 5 | 20 | 0.03 | 300 | 1.67481455 | 1.674524089 | 0.00029 |
| 5 | 20 | 0.03 | 400 | 1.83644975 | 1.836232943 | 0.000217 |
| 5 | 20 | 0.04 | 100 | 1.30015837 | 1.299073294 | 0.001085 |
| 5 | 20 | 0.04 | 200 | 1.52870254 | 1.530496426 | -0.00179 |
| 5 | 20 | 0.04 | 300 | 1.70297476 | 1.703803110 | -0.00083 |
| 5 | 20 | 0.04 | 400 | 1.86405054 | 1.864031107 | 1.94e-05 |
| 5 | 50 | 0.01 | 100 | 1.18927565 | 1.187522283 | 0.001753 |
| 5 | 50 | 0.01 | 200 | 1.42542186 | 1.425936064 | -0.00051 |
| 5 | 50 | 0.01 | 300 | 1.58943242 | 1.590085784 | -0.00065 |
| 5 | 50 | 0.01 | 400 | 1.71749227 | 1.717124282 | 0.000368 |
| 5 | 50 | 0.02 | 100 | 1.21903280 | 1.218425312 | 0.000607 |
| 5 | 50 | 0.02 | 200 | 1.45858793 | 1.458664405 | -7.65e-05 |
| 5 | 50 | 0.02 | 300 | 1.62540002 | 1.626017463 | -0.00062 |
| 5 | 50 | 0.02 | 400 | 1.75575511 | 1.756486107 | -0.00073 |
| 5 | 50 | 0.03 | 100 | 1.24959260 | 1.250077042 | -0.00048 |
| 5 | 50 | 0.03 | 200 | 1.49261945 | 1.491889115 | 0.00073 |
| 5 | 50 | 0.03 | 300 | 1.66232122 | 1.662327680 | -6.46e-06 |
| 5 | 50 | 0.03 | 400 | 1.79506981 | 1.796059281 | -0.00099 |
| 5 | 50 | 0.04 | 100 | 1.28071027 | 1.282320678 | -0.00161 |
| 5 | 50 | 0.04 | 200 | 1.52727786 | 1.525584772 | 0.001693 |
| 5 | 50 | 0.04 | 300 | 1.69993167 | 1.698968620 | 0.000963 |
| 5 | 50 | 0.04 | 400 | 1.83515656 | 1.835733552 | -0.00058 |
| 5 | 100 | 0.01 | 100 | 1.08327216 | 1.083649456 | -0.00038 |
| 5 | 100 | 0.01 | 200 | 1.34625802 | 1.346363740 | -0.00011 |
| 5 | 100 | 0.01 | 300 | 1.52509943 | 1.524190956 | 0.000908 |
| 5 | 100 | 0.01 | 400 | 1.66308255 | 1.663463668 | -0.00038 |
| 5 | 100 | 0.02 | 100 | 1.11624361 | 1.116886233 | -0.00064 |
| 5 | 100 | 0.02 | 200 | 1.38233784 | 1.382936541 | -0.0006 |
| 5 | 100 | 0.02 | 300 | 1.56311142 | 1.563357414 | -0.00025 |
| 5 | 100 | 0.02 | 400 | 1.70302782 | 1.703482716 | -0.00045 |
| 5 | 100 | 0.03 | 100 | 1.15073734 | 1.150850670 | -0.00011 |
| 5 | 100 | 0.03 | 200 | 1.41966918 | 1.420133153 | -0.00046 |
| 5 | 100 | 0.03 | 300 | 1.60232104 | 1.603157509 | -0.00084 |
| 5 | 100 | 0.03 | 400 | 1.74420378 | 1.744210134 | -6.35e-06 |
| 5 | 100 | 0.04 | 100 | 1.18627288 | 1.185612318 | 0.000661 |
| 5 | 100 | 0.04 | 200 | 1.45779091 | 1.457914190 | -0.00012 |
| 5 | 100 | 0.04 | 300 | 1.64234241 | 1.643585742 | -0.00124 |
| 5 | 100 | 0.04 | 400 | 1.78625600 | 1.785638490 | 0.000618 |
