# Supplementary material for: Artificial Neural Network analysis on the effect of mixed convection in triangular-shaped geometry using water-based Al2O3 nanofluid
Source: PLoS One. 2024 Sep 13;19(9):e0304826. doi: 10.1371/journal.pone.0304826 (PMC11398699; doi:10.1371/journal.pone.0304826)
Supplement: Attachment — Submitted filename: Response to the Reviewers.docx [file pone.0304826.s003.docx]

**Responses to the Reviewer Comments**

Manuscript No.:	PONE-D-24-08432R1

Manuscript Title:	Artificial Neural Network Analysis on the Effect of Mixed Convection in Triangular-shaped Geometry using Water-based Al_2_O_3_ Nanofluid

Author’s Name:	M. N. Hudha, Md. Jahid Hasan, T. Bairagi, A. K. Azad, M. M. Rahman

Thanks to the Reviewers’ valuable comments and suggestions. The manuscript has been revised according to the Reviewers’ comments and recommendations. The point by point response to the reviewers' comments is as follows:

**Respond to the comments of Reviewer 1**

\# Respect the following major corrections:

**Reviewer’s Comment 1**: Add SI units in a nomenclature table or within the text of the manuscript.

- Thank you for your comments and suggestions. The author appreciates your time and consideration in reviewing this article. The nomenclature has now been updated in the revised menuscripts and also it is given below:

**Nomenclature**

| *Β_0_* | *Magnetic field induction (T)* | *q_R_* | *Radiative heat flux* |
| --- | --- | --- | --- |
| *C_p_* | *Specific heat at constant pressure*  *(Jkg^-1^K^-1^)* | *P* | *Dimensionless pressure* |
| *H* | *Convective heat transfer coefficient (Wm^-2^K^-1^)* | *x, y* | *Dimensional cartesian coordinates* |
| *L* | *Length of the enclosure (m)* | *X, Y* | *Dimensionless cartesian coordinates* |
| *k* | *Thermal conductivity of the material (Wm^-1^K^-1^)* | *u, v* | *Dimensional velocity components (ms^-1^)* |
| *p* | *Dimensional pressure (Nm^-2^)* | *U, V* | *Dimensionless velocity components* |
| *ψ* | *Stream function* | *Re* | *Reynolds number* |
| *Pr* | *Prandtl number* | *Gr* | *Grashoff number* |
| *Ri* | *Richardson number* | *Ha* | *Hartmann number* |
| *Rd* | *Radiation parameter* |  |  |
|  | ***Greek Symbols*** |  |  |
| *α* | *Thermal diffusivity (m^2^s^-1^)* | *ω* | *Dimensionless rotation speed of the cylinder* |
| *β* | *Co-efficient of thermal expansion (K^-1^)* | *ν* | *Kinematic viscosity of the fluid (m^2^s^-1^)* |
| *ρ* | *Density of the material (kgm^-3^)* |  | ***Subscripts*** |
| *σ* | *Electrical conductivity (Ω^-1^m^-1^)* | *av* | *average* |
| *σ_e_* | *Stefan-Boltzmann constant* | *f* | *fluid* |
| *κ* | *Permeable parameter* | *h* | *hot* |
| *θ* | *Dimensional temperature (K)* | *c* | *cold* |
| *Θ* | *Dimensionless temperature* | *nf* | *nanofluid* |
| *ϕ* | *Solid volume fraction* | *p* | *solid particle* |
| *μ* | *Dynamic viscosity of the fluid (kgm^-1^s^-1^)* | *s* | *solid* |
| *ω_0_* | *Dimensional rotation speed of the cylinder (rad/s)* |  |  |

**Reviewer’s Comment 2:** The motivation part of the introduction should be improved with a clear specification of the considered novelties.

- Thank you for your comment. The motivation of the introduction has now been improved with a clear specification of the considered novelties as follows:

*“To this point, numerous studies have been conducted to investigate fluid flow and thermal performance in enclosed flows. However, there is a significant research gap that needs to be filled. This study aims to fill the research gap by using Artificial Neural Networks analysis in triangular-shaped cavities for magnetohydrodynamics (MHD) mixed convection with water/Al_2_O_3_ nanofluid. The introduction of ANN analysis attempts to offer a new method for comprehending the intricate relationships among fluid flow, heat transmission, and the factors that affect these cavities. This research addresses a gap in the existing literature and adds to the overall body of research by providing precise predictions and insights for improving triangular-shaped cavities under mixed convection with magnetohydrodynamics. This is especially pertinent for engineering applications and corresponds to the increasing interest in advanced computational methods for intricate fluid flow and heat transfer issues. Therefore, this study assesses the fluid flow, heat transfer, and conduct an Artificial Neural Network analysis for various Ri, Ha, and Re within the specified intervals of 0.01 ≤ Ri ≤ 5, 50 ≤ Re ≤ 200, and 0 ≤ Ha ≤ 100, respectively. Additionally, the study takes into account the three-variable nanofluid concentrations (1%, 3% and 4%) for the water-based Al_2_O_3_ nanofluid. The heat transfer and thermal science community will greatly benefit from the outcomes of this research.”*

**Reviewer’s Comment 3**: Enhance the introduction with the followng recent nanofluids' studies:
Influences of Blowing and Internal Heating Processes on Steady MHD Mixed Convective Boundary Layer Flows of Radiating Titanium Dioxide-Ethylene Glycol Nanofluids

- Generation of entropy on blood conveying silver nanoparticles embedded in curved surfaces

- Efficient Passive GDQLL Scrutinization of an Advanced Steady EMHD Mixed Convective Nanofluid Flow Problem via Wakif’s-Buongiorno Approach and Generalized Transport Laws

- Further insights into mixed convective boundary layer flows of internally heated Jeffery nanofluids: Stefan's blowing case study with convective heating and thermal radiation impressions

- Influences of blowing and internal heating processes on steady MHD mixed convective boundary layer flows of radiating titanium dioxide-ethylene glycol nanofluids

- A passive modeling strategy of steady MHD reacting flows for convectively heated shear-thinning/shear-thickening nanofluids over a horizontal elongating flat surface via Wakif’s-Buongiorno approach

- Effects of fractional derivative and heat source/sink on MHD free convection flow of nanofluids in a vertical cylinder: A generalized Fourier's law model

- Exploration of Multiple Transfer Phenomena within Viscous Fluid Flows over a Curved Stretching Sheet in the Co-Existence of Gyrotactic Micro-Organisms and Tiny Particles

- Water thermal enhancement in a porous medium via a suspension of hybrid nanoparticles: MHD mixed convective Falkner's-Skan flow

- Solutal effects on thermal sensitivity of casson nanofluids with comparative investigations on Newtonian (water) and non-Newtonian (blood) base liquid

- Thank you for suggesting these articles. These articles have been cited in the introduction section to improve the quality of the literature review. Please check the [3-7] and [11-13] citations.

*[3]	M. Alghamdi, A. Wakif, and T. Muhammad, “Efficient passive GDQLL scrutinization of an advanced steady EMHD mixed convective nanofluid flow problem via Wakif–Buongiorno approach and generalized transport laws,” Int. J. Mod. Phys. B, p. 2450418, 2024.*

*[4]	N. A. Shah et al., “Effects of fractional derivative and heat source/sink on MHD free convection flow of nanofluids in a vertical cylinder: A generalized Fourier’s law model, Case Stud. Therm. Eng., 28 (2021), 101518.” 2021.*

*[5]	M. Zaydan, A. Wakif, A. Alshehri, T. Muhammad, and R. Sehaqui, “A passive modeling strategy of steady MHD reacting flows for convectively heated shear-thinning/shear-thickening nanofluids over a horizontal elongating flat surface via Wakif’s-Buongiorno approach,” Numer. Heat Transf. Part A Appl., pp. 1–24, 2024.*

*[6]	K. Zhang, N. A. Shah, M. Alshehri, S. Alkarni, A. Wakif, and S. M. Eldin, “Water thermal enhancement in a porous medium via a suspension of hybrid nanoparticles: MHD mixed convective Falkner’s-Skan flow case study,” Case Stud. Therm. Eng., vol. 47, p. 103062, 2023.*

*[7]	A. Wakif, A. Alshehri, and T. Muhammad, “Influences of blowing and internal heating processes on steady MHD mixed convective boundary layer flows of radiating titanium dioxide‐ethylene glycol nanofluids,” ZAMM‐Journal Appl. Math. Mech. für Angew. Math. und Mech., p. e202300536, 2024.*

*[11]	J. Sharma, N. A. Ahammad, A. Wakif, N. A. Shah, J. D. Chung, and W. Weera, “Solutal effects on thermal sensitivity of casson nanofluids with comparative investigations on Newtonian (water) and non-Newtonian (blood) base liquids,” Alexandria Eng. J., vol. 71, pp. 387–400, 2023.*

*[12]	R. Zhang, M. Zaydan, M. Alshehri, C. S. K. Raju, A. Wakif, and N. A. Shah, “Further insights into mixed convective boundary layer flows of internally heating jeffery nanofluids: Stefan’s blowing case study with convective heating and thermal radiation impressions,” Case Stud. Therm. Eng., p. 104121, 2024.*

*[13]	K. Gangadhar, K. Shashidhar Reddy, M. Prameela, and A. Wakif, “Generation of entropy on blood conveying silver nanoparticles embedded in curved surfaces,” Proc. Inst. Mech. Eng. Part E J. Process Mech. Eng., p. 09544089231224523, 2024.*

**Reviewer’s Comment 4**: The authors have the possibility of integrating the hybrid nanofluids’ concepts instead of studying a monotype nanofluid. In this respect, it is recommended to add othe the effects and replace the present flow model with a realistic one.

- Thank you for your comment and suggestion. While it is true that several hybrid nanofluids have been invented, for the present paper, a water-based monotype nanofluid was used due to its wide usage in the industry and other applications. However, considering your comment, the authors will incorporate hybrid and ternary nanofluids in future studies based on this paper.

**Reviewer’s Comment 5**: The proposed solving strategy should be explained via a detailed flowchart and suitable references.

- Thank you for your comment and suggestion. The flow chart of ANN has been shown in Fig. 3.
- The illustrated ANN structure and Flow chart in **Fig. 3** comprises three layers: the input, hidden, and output layers.

**Fig. 3:** ANN model diagram (left) and ANN Flow chart (right)

**Reviewer’s Comment 6**: Validate your results.

- Thank you for your comment. The present study has been validated with the past literature of Khanafer and Chamkha [37] and Soomro et al. [38] The average Nusselt number has been seen to closely align with earlier investigations, exhibiting minimal error. In addition, a comparison for isotherms and streamlines have been added in the revised manuscrfor fixed values of Re = 400, Ri = 2 and Ha = 10 with the results of Xiong et al. [39]. Also, it is given below:

### ***3.3 Code Validation***

*The numerical model employed in this study has been validated against findings from previous literature. Specifically, comparisons were made between the Nusselt number, isotherm, and streamline contours to assess the accuracy of the results.* ***Table 3*** *compares the average Nu on the upper surface of the triangle with the values reported by Khanafer and Chamkha [37] and Soomro et al. [38] for Gr = 100 and Pr = 0.71. The average Nusselt number has been seen to closely align with earlier investigations, exhibiting minimal error.* ***Fig 2*** *depicts the comparison between Xiong et al. [39]’s study and present study in terms of isotherms(****Fig 2 (a))*** *and streamline contours (****Fig 2 (b))*** *at fixed value of Ri = 2, Re = 400 and Ha = 10 for right obstacle. It has been noted that the velocity and temperature distributions for both contours exhibit a high degree of similarity. Therefore, it can be confidently stated that the current study is well supported by prior literature. Further inquiries have been conducted in this study utilizing the identical code.*

**a**

**b**

Xiong et al. [39]

Present Study

**Fig. 2:** Model validation **(a)** Isotherm contours of Xiong et al. [39] and the present study, and **(b)** Streamlines contours of Xiong et al. [39] and the present study

**Reviewer’s Comment** **7**: Reinforce the results with proper physical explanations.

- Thank you for your comment and suggestion.The physical explanations behind the findings of the study have been added in the results and discussion section of the revised manuscript. Also, it is given below:

*“For a specific solid volume fraction condition, the fluid velocity streamline is found to be higher for higher Richardson number cases. This is because forced convection is dominant in such cases, leading to a higher velocity gradient.”*

*“It is observed that with the rise of the Richardson number, the temperature gradient increases, which is obvious. This is because the higher Richardson number ensures forced convection where velocity is involved.”*

*“The temperature of the fluid rises with the increase of nanoparticle concentrations. This is because the thermal conductivity is higher for a more concentrated nanofluid.”*

*“It has been noted that the Nu escalates as the concentration of nanofluid increases. This is because the higher concentration of nanofluid leads to an increase in thermal conductivity.”*

*“Reynolds number plays a vital role in augmenting the velocity gradient in the triangular-shaped cavity. It is found that the higher the Reynolds number, the greater the velocity streamline. This is because a higher Reynolds number denotes higher velocity.”*

*“It is observed from the plots that the Reynolds number has a positive effect on the fluid temperature. The higher the Reynolds number, the greater the temperature of the fluid is found. This is because higher fluid velocity ensures better heat dissipation inside the cavity, resulting in higher fluid temperature.”*

*“As a result, the highest heat transfer rate is found for the case of ϕ=4%, as before. The reason is that the more concentrated nanofluid is highly capable of transporting heat as the thermal conductivity is greater.”*

*“With the increase of the magnetic field, the velocity streamline gets reduced. This is because the fluid flow has been hindered due to magnetohydrodynamics.”*

*“It is observed that the heat transfer rate decreases with the increase in Ha. This is because the Hartmann number has a negative effect on fluid flow and heat transfer.”*

*“Furthermore, it is evident that the model worked incredibly well and that the predicted values were extremely precise because the difference between the targets and outputs was so little, as the error values were almost exactly zero.”*

**Respond to the comments of Reviewer 2**

\# Abstract section:

The abstract is well-written and effectively communicates the objectives, methodology, and key findings of the study. However, to enhance clarity and coherence, minor revisions are suggested:

**Reviewer’s Comment** **1**: Specify the objective of the study more explicitly in the first sentence.

- Thank you for your comments and suggestions. The author appreciates your time and consideration in reviewing this article. The first sentence has been added as follows:

*“The objective of the study is to investigate the fluid flow, heat transfer charecteristics applying Artificial Neural Networks (ANN) analysis in triangular-shaped cavities for the analysis of magnetohydrodynamics (MHD) mixed convection with varying fluid velocity of water/A_l2_O_3_ nanofluid.”*

**Reviewer’s Comment** **2**: Clarify the novelty and significance of employing ANN analysis in triangular-shaped cavities.

- Thank you for your comment. The clarification has been added in the following line:

*“No study has yet been conducted on this geometric configuration incorporating ANN analysis. Therefore, this study analyzes and predicts the complex interactions among fluid flow, heat transfer, and various influencing factors using ANN analysis.”*

**Reviewer’s Comment** **3**: Provide a brief explanation of the rationale behind selecting the Levenberg-Marquardt backpropagation technique for ANN.

- Thank you for your comment. A explanation of the rationale behind selecting the Levenberg Marquardt backpropagation technique for ANN has been given as follows:

*“The ANN model incorporates the Gauss-Newton method and the method of damped least squares, making it suitable for tackling complex problems with a high degree of non-linearity and uncertainty.”*

## ***Abstract***

*The objective of the study is to investigate the fluid flow and heat transfer characteristics applying Artificial Neural Networks (ANN) analysis in triangular-shaped cavities for the analysis of magnetohydrodynamics (MHD) mixed convection with varying fluid velocity of water/Al_2_O_3_ nanofluid. No study has yet been conducted on this geometric configuration incorporating ANN analysis. Therefore,, this study analyzes and predicts the complex interactions among fluid flow, heat transfer, and various influencing factors using ANN analysis. The process of finite element analysis was conducted, and the obtained results have been verified by previous literature. The Levenberg-Marquardt backpropagation technique was selected for ANN. Various values of the Richardson number (0.01 ≤ Ri ≤ 5), Hartmann number (0 ≤ Ha ≤ 100), Reynolds number (50 ≤ Re ≤ 200), and solid volume fraction of the nanofluid (ϕ = 1%, 3% and 4%) have been selected. The ANN model incorporates the Gauss-Newton method and the method of damped least squares, making it suitable for tackling complex problems with a high degree of non-linearity and uncertainty. The findings have been shown through the use of streamlines, isotherm plots, Nusselt numbers, and the estimated Nusselt number obtained by ANN. Increasing the solid volume fraction improves the rate of heat transmission for all situations with varying values of Ri, Re, and Ha. The Nusselt number is greater with larger values of the Ri and Re parameters, but it lessens for higher value of Ha. Furthermore, ANN demonstrates exceptional precision, as evidenced by the Mean Squared Error and R values of 1.05200e-6 and 0.999988, respectively.*

\# Introduction section:

**Reviewer’s Comment** **1**: The introduction provides a comprehensive overview of the existing literature in the field of fluid flow and heat transfer, which is commendable. However, there are instances where the flow of information could be improved for better clarity and organization. Consider structuring the introduction into subsections to delineate different research themes or methodologies, making it easier for readers to follow the progression of ideas.

- Thank you for your comment and suggestion. The introduction section has been carefully structured to cover various themes and methodologies related to this study. The first paragraph provides a focus on mixed convective flow in different enclosures and cavities. The second paragraph discusses the inclusion of magnetohydrodynamics in the study. The third section focuses on the use of nanofluids and nanoparticles to enhance heat transfer performance. Lastly, a section has been added to discuss the recent implementation of artificial neural networks. These four main methodologies and themes have been incorporated into this study, and therefore, the literature review in the introduction has been designed accordingly.

**Reviewer’s Comment** **2**: Ensure consistency in citation formatting throughout the introduction. Some citations are provided in brackets [1], while others are presented within the text (e.g., "Raza et al. [1]"). Adopt a consistent citation style throughout the introduction to enhance readability and professionalism.

- Thank you for your comment. In some cases, citations are placed within brackets at the end of the sentence (where no authors’ names were mentioned) to support the statement by referencing multiple papers or a single paper.

Additionally, in other instances, citations are presented within the text, such as "Raza et al. [1]", to specifically highlight a particular study. In that case, the citation has been put just after the name of the author.

**Reviewer’s Comment** **3**: Provide brief explanations of how each study contributes to the context of the current research, emphasizing their relevance to the research objectives.

- Thank you for your comment. The four sections of the introduction discuss the past literature which are relevant to this current study. The first paragraph discussed the investigation of mixed convective flow in diverse enclosures and cavities, which serves as a basis for comprehending the intricate fluid dynamics and heat transfer phenomena in restricted geometries. The second paragraph discussed the incorporation of Magnetohydrodynamics by examining the impact of magnetic fields on the movement of fluids and the transmission of heat. The third paragraph explained the study of nanofluids and nanoparticles to improve heat transfer efficiency by introducing nanoparticles into the base fluid. Lastly, the fourth paragraph talks about the use of Artificial Neural Networks (ANN), which presents a new method for analyzing and predicting fluid flow and heat transfer phenomena.

**Reviewer’s Comment** **4**: Consider refining the articulation of this research gap to clearly convey why addressing it is important and what specific knowledge voids it aims to fill within the broader research landscape.

- Thank you for for pointing this out. The novelty section of the introduction section has been edited following your comment as follows:

*“To this point, numerous studies have been conducted to investigate fluid flow and thermal performance in enclosed flows. However, there is a significant research gap that needs to be filled. This study aims to fill the research gap by using Artificial Neural Networks analysis in triangular-shaped cavities for magnetohydrodynamics (MHD) mixed convection with water/Al_2_O_3_ nanofluid. The introduction of ANN analysis attempts to offer a new method for comprehending the intricate relationships among fluid flow, heat transmission, and the factors that affect these cavities. This research addresses a gap in the existing literature and adds to the overall body of research by providing precise predictions and insights for improving triangular-shaped cavities under mixed convection with magnetohydrodynamics. This is especially pertinent for engineering applications and corresponds to the increasing interest in advanced computational methods for intricate fluid flow and heat transfer issues. Therefore, this study assesses the fluid flow, heat transfer, and conduct an Artificial Neural Network analysis for various Ri, Ha, and Re within the specified intervals of 0.01 ≤ Ri ≤ 5, 50 ≤ Re ≤ 200, and 0 ≤ Ha ≤ 100, respectively. Additionally, the study takes into account the three-variable nanofluid concentrations (1%, 3% and 4%) for the water-based Al_2_O_3_ nanofluid. The heat transfer and thermal science community will greatly benefit from the outcomes of this research.”*

**Reviewer’s Comment** **5**: Clearly state how the identified research gap motivates the current research and what specific aspects will be addressed in the subsequent sections of the paper.

- Thank you for your comment. The identified research gap and what specific aspects will be addressed in the subsequent sections of the paper has been discussed in the revised manuscript as follows:

*“To this point, numerous studies have been conducted to investigate fluid flow and thermal performance in enclosed flows. However, there is a significant research gap that needs to be filled. This study aims to fill the research gap by using Artificial Neural Networks analysis in triangular-shaped cavities for magnetohydrodynamics (MHD) mixed convection with water/Al2O3 nanofluid. The introduction of ANN analysis attempts to offer a new method for comprehending the intricate relationships among fluid flow, heat transmission, and the factors that affect these cavities. This research addresses a gap in the existing literature and adds to the overall body of research by providing precise predictions and insights for improving triangular-shaped cavities under mixed convection with magnetohydrodynamics. This is especially pertinent for engineering applications and corresponds to the increasing interest in advanced computational methods for intricate fluid flow and heat transfer issues. Therefore, this study assesses the fluid flow, heat transfer, and conduct an Artificial Neural Network analysis for various Ri, Ha, and Re within the specified intervals of 0.01 ≤ Ri ≤ 5, 50 ≤ Re ≤ 200, and 0 ≤ Ha ≤ 100, respectively. Additionally, the study takes into account the three-variable nanofluid concentrations (1%, 3% and 4%) for the water-based Al2O3 nanofluid. The heat transfer and thermal science community will greatly benefit from the outcomes of this research.”*

\# Mathematical and governing equation section:

**Reviewer’s Comment** **1**: It is suggested to provide description of terms such as density variation due to Boussineq approximation etc before incorporating into equation to provide readers a better understanding.

- Thank you for your comment. Based on the suggestion, the Boussineq approximation for density variation has been added in the revised manuscript. Also, it is given below:

*“Where due to significant temperature variations, density fluctuations related to temperature in the buoyancy term are approximated using a Boussinesq model. This means that density changes are assumed to follow a relationship with temperature* $-\rho_{0}\beta\left(T-T_{c}\right)g$*” .*

**Reviewer’s Comment** **2**: Also, few more steps leading to derivation of non-dimensional form could certainly benefit the readability and clarity of the paper

- Thank you for your comment. According to the reviewer’s comment a few more steps leading to derivation of non-dimentional form has been added in the revised manuscript. For the reviewer’s check, it is also given below:

**2.2.2 Dimensionless governing equations**

To formulate the Eqns. (1 - 4) in a dimensionless format about their initial and boundary conditions Eqn. (5). The dimensionless factors are:

$\left.\begin{matrix} X=\frac{x}{H},\text{ }Y=\frac{y}{H},\text{ }U=\frac{u}{v_{0}},\text{ }V=\frac{v}{v_{0}},\text{ }P=\frac{p}{\rho_{bf}v_{0}^{2}},\text{ }\theta=\frac{T-T_{c}}{T_{h}-T_{c}},\text{ } \\ \Pr=\frac{\upsilon_{bf}}{\alpha_{bf}},\text{ }Gr=\frac{g\beta_{bf}\left(T_{h}-T_{c}\right)H^{3}}{\upsilon_{bf}^{2}},\text{ }\mathrm{Re}=\frac{v_{0}H}{\upsilon_{bf}}\text{ },\text{ }Ri=\frac{Gr}{\mathrm{Re}^{2}},\text{ and }Ha=B_{0}H\sqrt{\frac{\sigma_{bf}}{\rho_{bf}\upsilon_{bf}}} \end{matrix}\right\}$ (6)

$\left.\begin{matrix} \therefore\frac{\partial u}{\partial x}=\frac{v_{0}}{H}\frac{\partial U}{\partial X},\frac{\partial u}{\partial y}=\frac{v_{0}}{H}\frac{\partial U}{\partial Y},\frac{\partial v}{\partial x}=\frac{v_{0}}{H}\frac{\partial V}{\partial X},\frac{\partial v}{\partial y}=\frac{v_{0}}{H}\frac{\partial V}{\partial Y}, \\ \frac{\partial p}{\partial x}=\frac{\rho_{bf}v_{0}^{2}}{H}\frac{\partial P}{\partial X},\frac{\partial p}{\partial y}=\frac{\rho_{bf}v_{0}^{2}}{H}\frac{\partial P}{\partial Y},\frac{\partial T}{\partial x}=\frac{\Delta T}{H}\frac{\partial\theta}{\partial X},\frac{\partial T}{\partial y}=\frac{\Delta T}{H}\frac{\partial\theta}{\partial Y} \end{matrix}\right\}$ (7)

$\left.\begin{matrix} \therefore\frac{\partial^{2}u}{\partial x^{2}}=\frac{v_{0}}{H^{2}}\frac{\partial^{2}U}{\partial X^{2}},\frac{\partial^{2}u}{\partial y^{2}}=\frac{v_{0}}{H^{2}}\frac{\partial^{2}U}{\partial Y^{2}},\frac{\partial^{2}v}{\partial x^{2}}=\frac{v_{0}}{H^{2}}\frac{\partial^{2}V}{\partial X^{2}}, \\ \frac{\partial^{2}v}{\partial y^{2}}=\frac{v_{0}}{H^{2}}\frac{\partial^{2}V}{\partial Y^{2}},\frac{\partial^{2}T}{\partial x^{2}}=\frac{\Delta T}{H^{2}}\left(\frac{\partial^{2}\theta}{\partial X^{2}}\right),\frac{\partial^{2}T}{\partial y^{2}}=\frac{\Delta T}{H^{2}}\left(\frac{\partial^{2}\theta}{\partial Y^{2}}\right) \end{matrix}\right\}$ (8)

Applying the Eqns. (6) –(8), the continuity, momentum, and energy equations- the dimensionless equations are as follows:

$\frac{\partial U}{\partial X}+\frac{\partial V}{\partial Y}=0$ (9)

$U\frac{\partial U}{\partial X}+V\frac{\partial U}{\partial Y}=-\frac{\rho_{bf}}{\rho_{nf}}\frac{\partial P}{\partial X}+\frac{\upsilon_{nf}}{\upsilon_{bf}}\frac{1}{\mathrm{Re}}\left(\frac{\partial^{2}U}{\partial X^{2}}+\frac{\partial^{2}U}{\partial Y^{2}}\right)$ (10)

$U\frac{\partial V}{\partial X}+V\frac{\partial V}{\partial Y}=-\frac{\rho_{bf}}{\rho_{nf}}\frac{\partial P}{\partial Y}+\frac{\upsilon_{nf}}{\upsilon_{bf}}\frac{1}{\mathrm{Re}}\left(\frac{\partial^{2}V}{\partial X^{2}}+\frac{\partial^{2}V}{\partial Y^{2}}\right)+\frac{\left(\rho\beta\right)_{nf}}{\rho_{nf}\beta_{bf}}Ri\theta-\frac{\rho_{bf}}{\rho_{nf}}\frac{\sigma_{nf}}{\sigma_{bf}}\frac{Ha^{2}}{\mathrm{Re}}V$ (11)

$U\frac{\partial\theta}{\partial X}+V\frac{\partial\theta}{\partial Y}=\frac{\alpha_{nf}}{\alpha_{bf}}\frac{1}{\mathrm{RePr}}\left(\frac{\partial^{2}\theta}{\partial X^{2}}+\frac{\partial^{2}\theta}{\partial Y^{2}}\right)$ (12)

Then, the dimensionless boundary conditions for this specified problem are

$\left.\begin{matrix} \text{At the vertical boundary: }U=0,\text{ }V=1,\text{ }\frac{\partial\theta}{\partial N}=0 \\ \text{At the horizontal boundary: }U=0,\text{ }V=0, \theta=\sin\left(\pi x\right)\text{ } \\ \text{At the curve boundary: }U=0,\text{ }V=0,\text{ }\theta=\theta_{c} \end{matrix}\right\}$ (13)

\# Conclusion section:

**Reviewer’s Comment** **1**: The conclusion could benefit from clearer structuring to enhance readability. Consider breaking it down into subsections corresponding to the main findings discussed, such as the impact of nanofluid concentration, forced convection, fluid velocity, and magnetohydrodynamics. This would make it easier for readers to navigate and comprehend the key findings.

- Thank you for your comment and suggestion. Following your suggestion, the conclusion findings have been broken down into subsections corresponding to the main findings in the revised manuscript and also, it is given as follows:

## ***5. Conclusions***

*This investigation was conducted to study the fluid flow and thermal performance in a triangular-shaped cavity using a nanofluid consisting of Al_2_O_3_ particles suspended in water. The study considered the effects of mixed convection, magnetohydrodynamics (MHD), and varying Reynolds numbers. Various values of the Richardson number (Ri), Hartmann number (Ha), Reynolds number (Re), and solid volume percentage of the nanofluid (ϕ) were examined. The Levenberg-Marquardt backpropagation approach was employed to do an analysis utilizing Artificial Neural Network (ANN).*

*The primary discoveries of the investigation are as follows:*

- *The concentration of nanofluid has a major impact on the rate of heat transfer, with higher concentrations of nanoparticles leading to improved heat transfer. A concentration of 4% shown superior thermal performance in all cases.*
- *In cases when forced convection is the dominant factor, there is an increase in thermal transfer within the cavity. The Nusselt number reached its maximum value of 1.85 at Ri = 4, whilst the heat transfer rate was at its minimum when Ri = 1 with the value of 1.72. Furthermore, greater Richardson number values resulted in elevated temperature gradients.*
- *The rate of heat transmission is directly influenced by the velocity of the fluid. The Nusselt number reached its maximum value of 1.7 at a Reynolds number of 400 and a concentration of 4%, whilst the minimum heat transfer rate (Nu=1.6) was observed at a Reynolds number of 100. Increased velocities were correlated with larger streamline velocity and temperature gradients.*
- *Magnetohydrodynamics (MHD) has a detrimental effect on the transmission of heat and the movement of fluid in the cavity. Higher Hartmann numbers lead to reduced fluid flow, decreased temperature gradients, and lower Nusselt numbers. The Nusselt number reached its maximum value of 1.19 at Ha = 100, but its minimum value (0.9) was observed at Ha = 400.*
- *The analysis using Artificial Neural Networks (ANN) demonstrated exceptional accuracy, as seen by the low Mean Squared Error (MSE= 1.05200e-6) and high R values (0.999988) for the validation data. The low error rate further confirms the efficacy of ANN in accurately forecasting the heat transfer performance of the triangular-shaped cavity under study.*

*This study helps fill the research gap by offering valuable insights into the fluid flow and thermal performance within triangular-shaped cavities, utilizing nanofluids and employing artificial neural network (ANN) analysis. This study improves our understanding of improving the thermal behavior of systems by examining the effects of nanofluid concentration, forced convection, fluid velocity, and MHD.*

*The practical applications of these results have far-reaching ramifications for the design and optimization of thermal devices and systems. The findings of this study can be utilized to increase the efficiency of heat transmission and boost the performance of triangular-shaped cavities in different engineering applications.*

*To enhance our comprehension of fluid flow and thermal performance, it is advisable to investigate alternative nanofluid compositions and cavity configurations in future studies. Furthermore, conducting research on the impacts of other boundary conditions and analyzing more intricate geometrical configurations will yield significant knowledge. Moreover, investigating sophisticated artificial neural network structures and optimization techniques might improve the precision and effectiveness of ANN analysis for comparable systems.*

**Reviewer’s Comment** **2:** Provide more specific details regarding the findings, such as numerical values of Nusselt numbers, temperature gradients, and fluid flow velocities for each parameter combination investigated. This would provide readers with a clearer understanding of the magnitude of the observed effects and facilitate comparison with previous studies.

- Thank you for pointing this out. For the clear understanding of the reader the magnitude of the observed effects the specific details with the numerical values have been added in the revised conclusion section.

**Reviewer’s Comment** **3:** Connect the findings discussed in the conclusion more explicitly with the existing literature reviewed in the introduction. Highlight how the observed trends and results contribute to addressing the identified research gap and advancing our understanding of fluid flow and thermal performance in triangular-shaped cavities using nanofluids and ANN analysis.

- Thank you for your comment and suggestion. The findings have been connected by discussing the conclusion more explicitly by highlighting the observed trends and results, addressing the identified research gap, and advancing the understanding of the fluid flow and thermal performance in triangular-shaped cavities using nanofluids and ANN analysis as follows:

*“This study helps fill the research gap by offering valuable insights into the fluid flow and thermal performance within triangular-shaped cavities, utilizing nanofluids and employing artificial neural network (ANN) analysis. This study improves our understanding of improving the thermal behavior of systems by examining the effects of nanofluid concentration, forced convection, fluid velocity, and MHD.”*

**Reviewer’s Comment** **4:** Discuss the broader implications of the findings in terms of their significance for practical applications and future research directions. Consider addressing how the insights gained from this study can inform the design and optimization of thermal devices and systems, as well as potential areas for further investigation.

- Thank you for your comments and suggestions. The broader implications of the findings in terms of their significance for practical applications and future research directions have been discussed along with addressing how the insights gained from this study can inform the design and optimization of thermal devices and systems, as well as potential areas for further investigation as follows:

*“The practical applications of these results have far-reaching ramifications for the design and optimization of thermal devices and systems. The findings of this study can be utilized to increase the efficiency of heat transmission and boost the performance of triangular-shaped cavities in different engineering applications.*

*To enhance our comprehension of fluid flow and thermal performance, it is advisable to investigate alternative nanofluid compositions and cavity configurations in future studies. Furthermore, conducting research on the impacts of other boundary conditions and analyzing more intricate geometrical configurations will yield significant knowledge. Moreover, investigating sophisticated artificial neural network structures and optimization techniques might improve the precision and effectiveness of ANN analysis for comparable systems.”*

**Respond to the comments of the Editor**

Thank you for submitting your manuscript to PLOS ONE. After careful consideration, we feel that it has merit but does not fully meet PLOS ONE’s publication criteria as it currently stands. Therefore, we invite you to submit a revised version of the manuscript that addresses the points raised during the review process.

Please submit your revised manuscript by May 11 2024 11:59PM.

- The authors appreciate the Editor’s suggestions and the consideration. The authors carefully revised the manuscript in response to the reviewers' remarks, taking into account all of the points mentioned. The authors believe that the quality of the paper has been improved enough for it to be accepted in its revised version.
