# Supplementary material for: Artificial Neural Network analysis on the effect of mixed convection in triangular-shaped geometry using water-based Al2O3 nanofluid
Source: PLoS One. 2024 Sep 13;19(9):e0304826. doi: 10.1371/journal.pone.0304826 (PMC11398699; doi:10.1371/journal.pone.0304826)
Supplement: Attachment — Submitted filename: Reviewer comments.pdf [file pone.0304826.s002.pdf]

Abstract section:

The abstract is well-written and effectively communicates the objectives, methodology, and key findings of the study. However, to enhance clarity and coherence, minor revisions are suggested:

1. Specify the objective of the study more explicitly in the first sentence.
2. Clarify the novelty and significance of employing ANN analysis in triangular-shaped cavities.
3. Provide a brief explanation of the rationale behind selecting the Levenberg-Marquardt backpropagation technique for ANN.

Introduction section:

1. The introduction provides a comprehensive overview of the existing literature in the field of fluid flow and heat transfer, which is commendable. However, there are instances where the flow of information could be improved for better clarity and organization. Consider structuring the introduction into subsections to delineate different research themes or methodologies, making it easier for readers to follow the progression of ideas.
2. Ensure consistency in citation formatting throughout the introduction. Some citations are provided in brackets [1], while others are presented within the text (e.g., "Raza et al. [1]"). Adopt a consistent citation style throughout the introduction to enhance readability and professionalism.
3. Provide brief explanations of how each study contributes to the context of the current research, emphasizing their relevance to the research objectives.
4. Consider refining the articulation of this research gap to clearly convey why addressing it is important and what specific knowledge voids it aims to fill within the broader research landscape.
5. Clearly state how the identified research gap motivates the current research and what specific aspects will be addressed in the subsequent sections of the paper.

Mathematical and governing equation section:

1. It is suggested to provide description of terms such as density variation due to Boussineq approximation etc before incorporating into equation to provide readers a better understanding.
2. Also, few more steps leading to derivation of non-dimensional form could certainly benefit the readability and clarity of the paper

Conclusion section:

1. The conclusion could benefit from clearer structuring to enhance readability. Consider breaking it down into subsections corresponding to the main findings discussed, such as the impact of nanofluid concentration, forced convection, fluid velocity, and

magnetohydrodynamics. This would make it easier for readers to navigate and comprehend the key findings.

2. Provide more specific details regarding the findings, such as numerical values of Nusselt numbers, temperature gradients, and fluid flow velocities for each parameter combination investigated. This would provide readers with a clearer understanding of the magnitude of the observed effects and facilitate comparison with previous studies.

3. Connect the findings discussed in the conclusion more explicitly with the existing literature reviewed in the introduction. Highlight how the observed trends and results contribute to addressing the identified research gap and advancing our understanding of fluid flow and thermal performance in triangular-shaped cavities using nanofluids and ANN analysis.

4. Discuss the broader implications of the findings in terms of their significance for practical applications and future research directions. Consider addressing how the insights gained from this study can inform the design and optimization of thermal devices and systems, as well as potential areas for further investigation.
